# Lateral migration resistance of screw is essential in evaluating bone screw stability of plate fixation

**DOI:** 10.1038/s41598-021-91952-3

**Published:** 2021-06-15

**Authors:** Xiaoreng Feng, Weichen Qi, Teng Zhang, Christian Fang, Hongfeng Liang, Bin Chen, Frankie Leung

**Affiliations:** 1 Department of Orthopaedics and Traumatology, Queen Mary Hospital, the University of Hong Kong, Pok Fu Lam, Hong Kong, SAR China; 2 Division of Orthopaedics and Traumatology, Department of Orthopaedics, Nanfang Hospital, Southern Medical University, Guangzhou, 510515 China; 3 Yangjiang People’s Hospital, Yangjiang, 529500 China

**Keywords:** Translational research, Mechanical engineering

## Abstract

Conventional evaluation of the stability of bone screws focuses on pullout strength, while neglecting lateral migration resistance. We measured pullout strength and lateral migration resistance of bone screws and determined how these characteristics relate to screw stability of locking plate (LP) and dynamic compression plate (DCP) fixation. Pullout strength and lateral migration resistance of individual bone screws with buttress, square, and triangular thread designs were evaluated in polyurethane foam blocks. The screw types with superior performance in each of these characteristics were selected. LP and DCP fixations were constructed using the selected screws and tested under cyclic craniocaudal and torsional loadings. Subsequently, the association between individual screws’ biomechanical characteristics and fixation stability when applied to plates was established. Screws with triangular threads had superior pullout strength, while screws with square threads demonstrated the highest lateral migration resistance; they were selected for LP and DCP fixations. LPs with square-threaded screws required a larger force and more cycles to trigger the same amount of displacement under both craniocaudal and torsional loadings. Screws with triangular and square threads showed no difference in DCP fixation stability under craniocaudal loading. However, under torsional loading, DCP fixation with triangular-threaded screws demonstrated superior fixation stability. Lateral migration resistance is the primary contributor to locking screw fixation stability when applied to an LP in resisting both craniocaudal and torsional loading. For compression screws applied to a DCP, lateral migration resistance and pullout strength work together to resist craniocaudal loading, while pullout strength is the primary contributor to the ability to resist torsional loading.

## Introduction

The use of bone screws to stabilize orthopedic plates is a widely applied technique in fracture treatment^1^. Plates and screws can be secured to fractured long bones of arms and legs, thus providing a stable environment for bone healing. Locking and compression screws are the two most commonly used types of screws in the treatment of long bone fractures.

Despite favorable clinical results, the number of clinically reported screw failures is high^2–5^. Hip fracture screw failure rates vary between 3 and 5%^2,3^, while those for proximal humeral fractures vary between 15 and 40%^4,5^. It has been suggested that at least one million pedicle screw failures occur annually worldwide due to loosening and/or migration; and while some failures do not have significant clinical consequences, some may need immediate and costly revision surgery^6^.

To overcome some of these difficulties, cement augmentation and improved screw design have been used to increase the holding power of bone screws^7–9^; however, previous studies have only evaluated holding power in pure axial pullout experiments^10–15^, that represents the exclusive standardized method to test the anchorage strength of medical bone screws^16^. In research of the pedicle screw, pullout strength has been found to be an unsuitable predictor of screw loosening, because it does not simulate physiological loading conditions^17,18^. Cyclic fatigue loading tests, also referred to as toggle tests, are considered more suitable for pedicle screw testing^17,19,20^. The loading of the human arms and/or legs during daily activity is highly complex, and plate/screw constructs experience both vertical (screw lateral migration) and horizontal (screw pullout) forces. Therefore, using a pullout test alone to evaluate bone screw stability cannot realistically simulate the in vivo loading conditions of plate/screw constructs to predict its real fixation strength. Our previous research on screw loosening mechanisms revealed that high radial stress around the screw is the primary reason for screw loosening^21^. Lateral migration resistance, the ability to resist lateral movements of the screw during physiological loading of the bone-implant construct, has great potential to become an important performance metric when evaluating the fixation stability of a screw design. Therefore, lateral migration resistance, another important characteristic of bone screws, should also be considered when evaluating their fixation stability for long bone fracture plate fixation.

Locking screws and compression screws are under different loading patterns during physiological loading because of different plate/screw interfaces (locking and non-locking)^22^. Which characteristic of bone screws (axial pullout strength or lateral migration resistance) primarily determines the fixation stability of each screw type remains unclear, and this knowledge would be useful in guiding us to apply appropriate tests to the development of new screw designs and the validation of existing screws. Therefore, the aim of this study was to clarify the association between the characteristics of individual bone screws and the fixation stability of plate/screw constructs. We hypothesized that lateral migration resistance of bone screws has a higher association with the fixation stability of plate/screw constructs than pullout strength.

## Materials and methods

**Figure 1:**
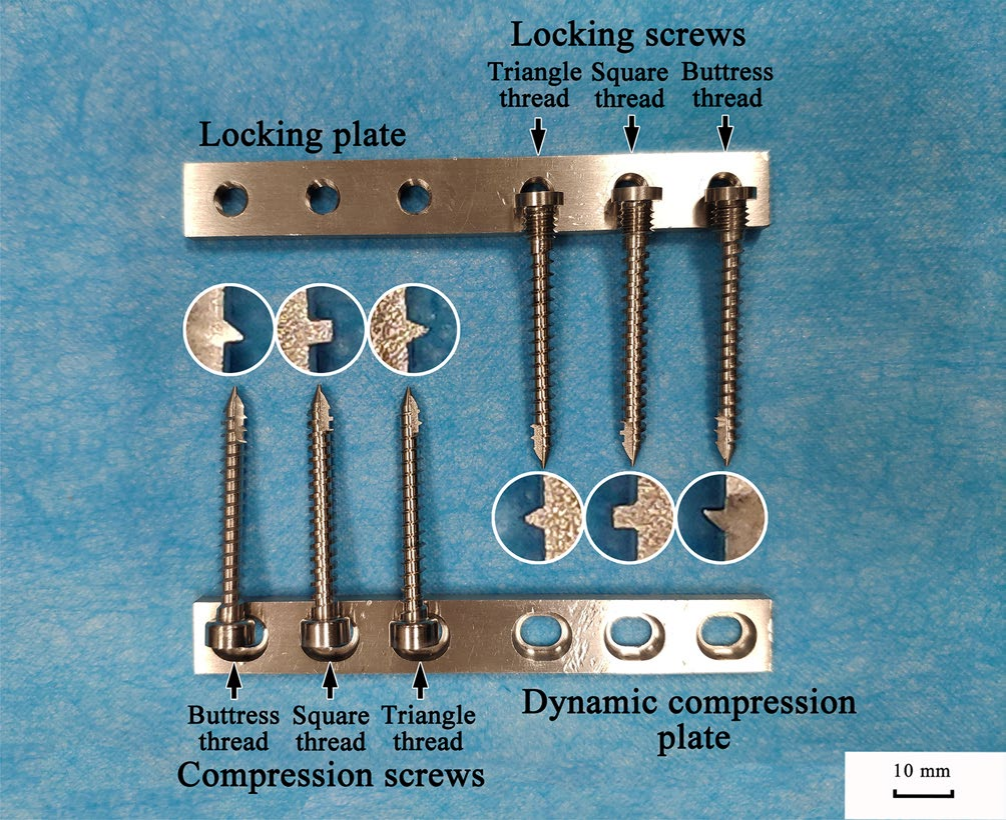
Screws and corresponding plates for the biomechanical tests in this study.

Locking screws and compression screws with three different kinds of thread designs (buttress thread, square thread, and triangle thread) were manufactured from 316 LVM stainless steel. All screws were self-tapping screws with a thread pitch of 2.0 mm, major and minor diameters of 4.5 mm and 3.2 mm, respectively, a screw shaft length of 38 mm, and had the same cutting flute design. The 4.5 mm screws were used to be compatible with the standard instrumentation for the 4.5 mm AO cortical screw by relying on identical 3.2 mm diameter drill bits. Locking plates (LP) and dynamic compression plates (DCP) were specially designed for the above locking and compression screws and were manufactured from 316 LVM stainless steel (Fig. 1). Both plate types were 12 mm wide, 95 mm long, 4 mm thick, and had 15 mm hole-spacing, representing plates for human long bone fracture repair. Solid rigid polyurethane foam blocks with a density of 0.32 g/cm^3^, (Sawbones, Vashon, Washington, USA) adhering to ASTM F-1839–08, were used^23^. This density was chosen because it has similar biomechanical strength to normal cancellous bone, which has been validated by screw pullout testing in previous studies^24–26^.

### Individual screw biomechanical characteristics

Two biomechanical characteristics for each kind of screw, axial pullout strength and lateral migration resistance, were tested in this study. Since the biomechanical characteristics of different screw types are largely influenced by the threaded screw shaft, which is the same in both screw types, these characteristics were only measured in compression screws.

**Figure 2:**
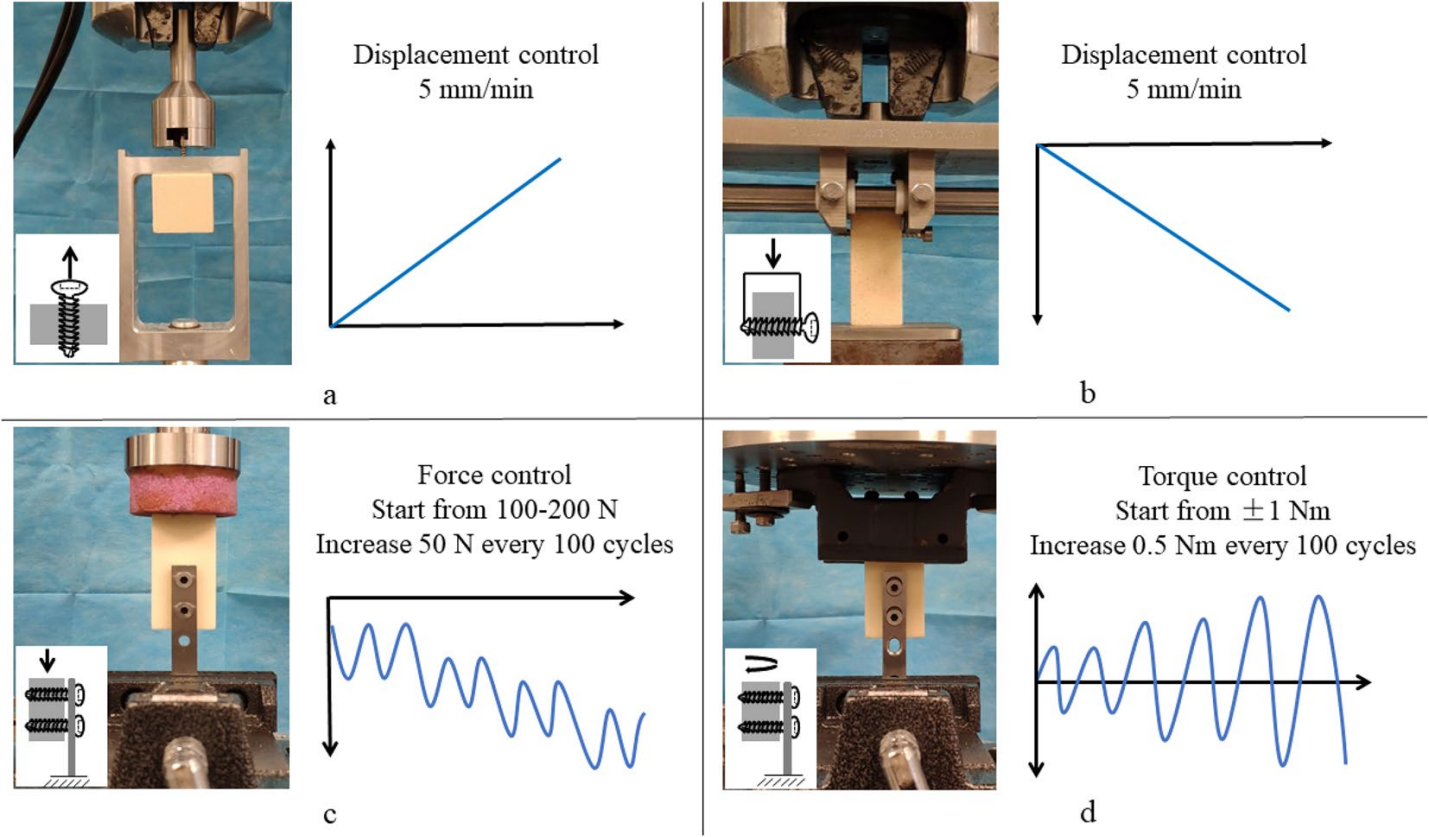
Experimental setups used in this study. (**a**) Individual screw axial pullout test; (**b**) individual screw lateral migration test; screw stability of plate fixation test under (**c**) craniocaudal loading and (**d**) torsional loading.

For the axial pullout test (Fig. 2a), 15 polyurethane foam cubes were tested. Polyurethane foam blocks were cut into cubes measuring 40 × 40 × 40 mm. A pilot hole of 3.2 mm in diameter was made all the way through the center of each polyurethane foam cube with a 3.2 mm drill bit by a drill press. The foam cubes were randomly divided into three groups for use with the three different thread types. Five screws per group were screwed 25 mm deep into the pre-drilled pilot holes i.e., a 25 mm long section of thread was anchored vertically into the foam cube. Each screw/ foam cube construct was mounted onto the load cell (0–1000 N) of an MTS 858 Mini Bionix (MTS, Inc., Minnesota, USA) hydraulic loading machine with a custom-made jig to ensure strict axial tension on the screw. The screws were extracted 10 mm from their starting point under displacement control at a rate of 5 mm/min according to published standards^16^.

For the lateral migration test (Fig. 2b), 15 polyurethane foam rectangular blocks were tested. The polyurethane foam blocks were cut into rectangular blocks measuring 20 × 20 × H50 mm. A 20 mm long pilot hole of 3.2 mm in diameter was drilled completely through each rectangular block along one of the 20 mm axes with a 3.2 mm drill bit by a drill press. Five screws per group were each screwed into the pilot hole until the cutting flute exited the rectangular block. A 20 mm long section of thread was anchored horizontally into the rectangular block for all screws. The rectangular block was fixed while the two ends of the screw were compressed vertically at 5 mm/min to 10 mm displacement using an MTS 858 Mini Bionix (MTS, Inc., Minnesota, USA) hydraulic loading machine.

The force versus displacement data were collected at 10 Hz as the screw pulled out axially or migrated laterally through the foam block. A force–displacement curve was plotted, and stiffness, yield force, and ultimate force of the curve were calculated. Stiffness was calculated as the slope of a best-fit line for the linear region of the force–displacement curve. Yield force was determined using a 0.05 mm offset parallel to the stiffness^27^. Ultimate force for the axial pullout test was the force measured at the peak of the curve, and for the lateral migration test, it was defined as the force at 5 mm of lateral migration. Normality of data distribution was checked and proved with Shapiro–Wilk test in order to apply parametric statistics. One-way ANOVA with Tukey's post-hoc test was used to compare stiffness, yield force, and ultimate force from different groups. Values of p < 0.05 were considered significant for all hypothesis tests.

The screw types with the highest axial pullout strength, and the highest lateral migration resistance were selected for testing screw stability when applied to a plate.

### Screw stability of plate fixation

For the screw stability test when applied to a plate, polyurethane foam blocks were cut into rectangular blocks measuring 30 × 30 × H60 mm to represent the proximal fragment of a long bone fracture. All specimens were pre-drilled in two planned locations with a 3.2 mm drill bit by a drill press. One end of the plate was secured to the rectangular foam block using two of the same type of screws to build the fracture plate fixation construction, and the other end of the plate that did not attach to the bone was fixed vertically on the load cell (0–1000 N) (Fig. 2c,d). The fixation stability of locking screws and compression screws when applied to a plate were tested in this study, so LP and DCP were used with their respective screw types. Each plate fixation type had two groups, plates fixed by screws with the highest axial pullout strength, and plates fixed by screws with the highest lateral migration resistance. Each group had five replicate models. In the LP fixation model, the plate was offset by 2 mm from the bone^28^ and the locking screws were tightened with a torque control screw driver (DePuy Synthes, Raynham, MA). In the DCP fixation model, all the compression screws were tightened with a maximum torque of 1 Nm (50% of the stripping torque)^29,30^ by the same surgeon manually with a calibrated torque wrench (Kanon DPSK-N20; Nakamura, Tokyo, Japan).

A loading force was applied to the upper surface of the foam block using an MTS Mini Bionix system (Fig. 2c, d). Since craniocaudal loading and torsional loading are the two main loading forms for the long bones of the arms and legs during daily activity, they were used to test the fixation stability of the screws. For cyclic craniocaudal loading, the force applied started at 100–200 N and increased by 50 N every 100 cycles with a frequency of 1 Hz (Fig. 2c). Cyclic craniocaudal loading of the foam block terminated once the MTS detected a total craniocaudal displacement of 5 mm. The force and number of cycles required to achieve a 1, 2, 3, 4, and 5 mm displacement were recorded for each screw type. For cyclic torsional loading, the torque applied started at ± 1 Nm and increased by 0.5 Nm every 100 cycles with a frequency of 1 Hz (Fig. 2d). Cyclic torsional loading of the foam block terminated once the MTS detected a total torsional angle of 10 degrees. The torque and number of cycles required to achieve 2, 4, 6, 8, and 10 degrees of torsion were recorded for each screw type. Normality of data distribution was checked and proved with Shapiro–Wilk test in order to apply this parametric statistics. The difference in fixation stability between the two screw types was analyzed using the independent samples Student’s t-test. Values of p < 0.05 were considered significant for all hypothesis tests.

### Association between individual screws’ biomechanical characteristics and stability of plate fixation

Comprehensively analyzing the results of the individual screws’ biomechanical characteristic tests and the test of the screws’ stability when applied to a plate to clarify the association between the axial pullout strength and lateral migration resistance of bone screws and the screw stability of LP and DCP fixation under certain loading patterns.

## Results

### Individual screws’ biomechanical characteristics

**Figure 3:**
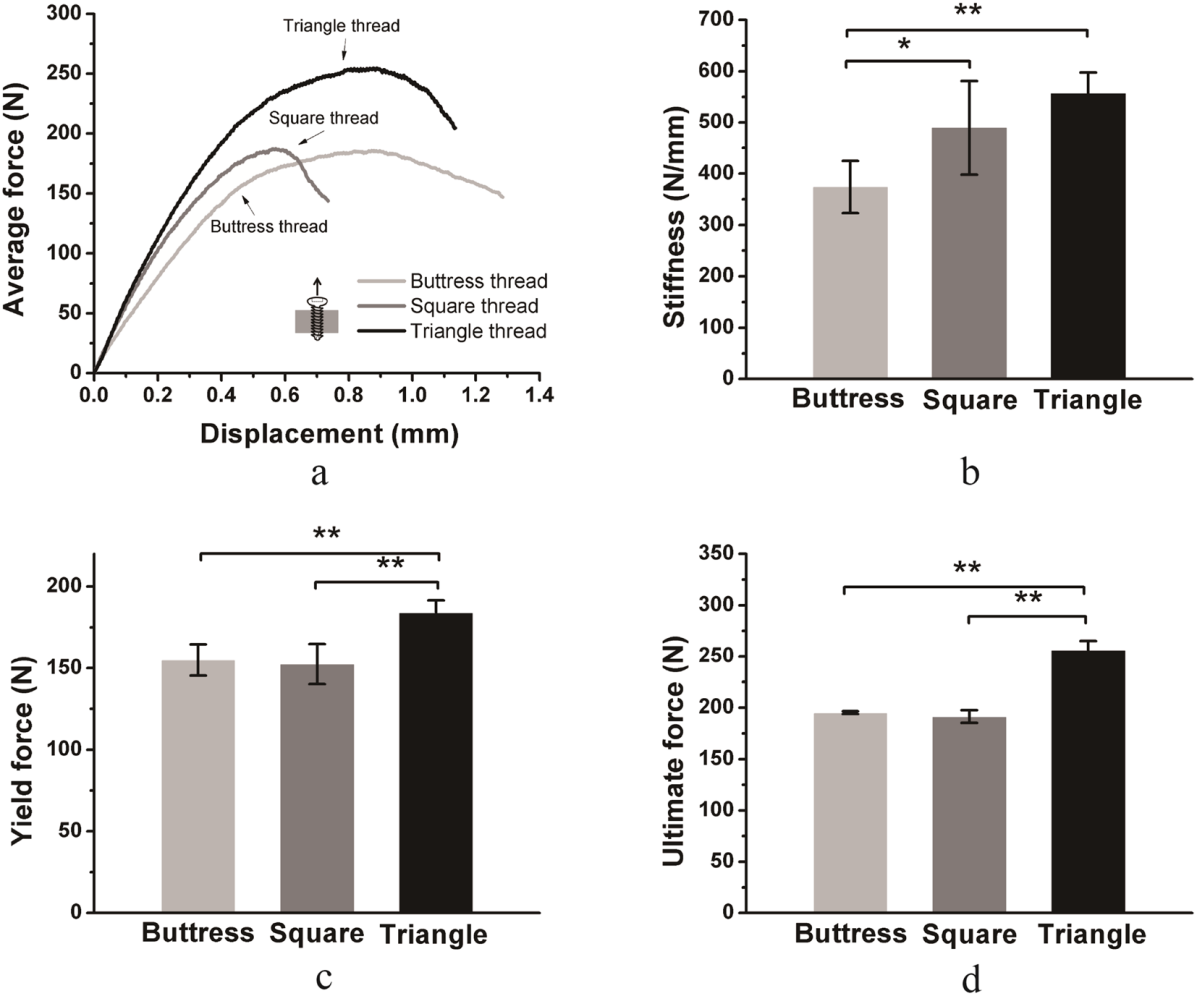
Comparison of the axial pullout strength of screws with different threads. Mean values ± standard deviation of the measured values are presented (N = 5) (one-way ANOVA, *p < 0.05, **p < 0.01). (**a**) Force–displacement curves of the pullout test. (**b**–**d**) Stiffness, yield force, and ultimate force of the force–displacement curve.

Representative force–displacement curves of the axial pullout tests were shown in Fig. 3a. In these curves, only the data up to the point where force decreased to 20% below the maximum was reserved in order to show the most important information. Triangle threaded screws performed better than buttress threaded or square threaded screws under axial pullout. The three parameters of stiffness, yield force, and ultimate force, were shown in Fig. 3b–d. The stiffness values of buttress threaded, square threaded, and triangle-threaded screws were 373.6 ± 50.8, 489.2 ± 91.5, and 556.8 ± 40.9 N/mm, respectively. The stiffness values of triangle threaded and square threaded screws were significantly higher than that of buttress-threaded screws (p < 0.05) (Fig. 3b). The yield forces of buttress threaded, square threaded, and triangle-threaded screws were 154.9 ± 9.6, 152.4 ± 12.3, and 184.0 ± 7.5 N, respectively. The ultimate forces of buttress threaded, square threaded, and triangle threaded screws were 195.1 ± 1.4, 191.3 ± 6.1, and 256.2 ± 8.9 N, respectively. Triangle threaded screws had significantly higher yield force and ultimate force than buttress threaded and square threaded screws (p < 0.01) (Fig. 3c, d). In general, screws with triangle threads had better axial pullout strength than screws with buttress threads or square threads.

**Figure 4:**
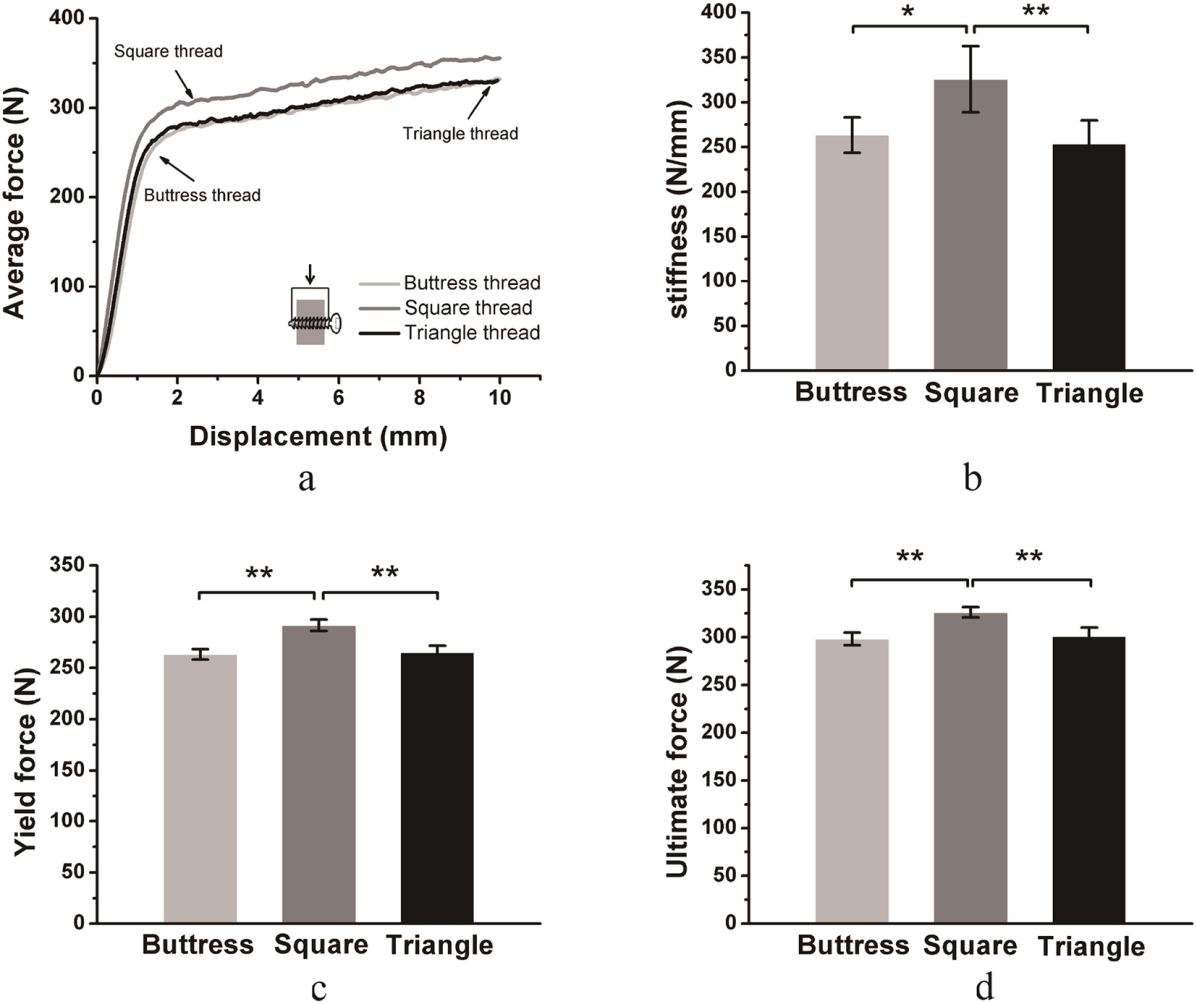
Comparison of the lateral migration resistance of screws with different threads. Mean values ± standard deviation of the measured values are presented (N = 5) (one-way ANOVA, *p < 0.05, **p < 0.01). (**a**) Force–displacement curves of the lateral migration resistance test. (**b**–**d**) Stiffness, yield force, and ultimate force of the force–displacement curve.

The force–displacement curve of the lateral migration test was shown in Fig. 4a. Square-threaded screws performed better than buttress threaded or triangle threaded screws in the lateral migration test. The three parameters of stiffness, yield force, and ultimate force, were shown in Fig. 2b–d. The stiffness values of buttress threaded, square threaded, and triangle-threaded screws were 263.2 ± 19.8, 325.6 ± 36.7 and 253.3 ± 26.2 N/mm, respectively. The stiffness of square threaded screws was significantly higher than buttress threaded or triangle threaded screws (p < 0.05) (Fig. 4b). The yield forces of buttress threaded, square threaded, and triangle-threaded screws were 263.2 ± 5.2, 291.6 ± 5.5 and 265.1 ± 6.4 N, respectively. The ultimate forces of the buttress threaded, square threaded, and triangle threaded screws were 298.2 ± 6.5, 326.0 ± 5.3 and 300.7 ± 9.4 N, respectively. Square threaded screws had significantly higher yield force and ultimate force than buttress threaded or triangle threaded screws (p < 0.01) (Fig. 4c,d). In general, screws with square threads had better lateral migration resistance than screws with buttress threads and triangle threads.

The above results showed that screws with triangle threads were superior in axial pullout strength, whereas screws with square threads were superior in lateral migration resistance. Therefore, screws with triangle threads and square threads were selected for the screw stability of plate fixation test.

### Screws’ stability of plate fixation

**Figure 5:**
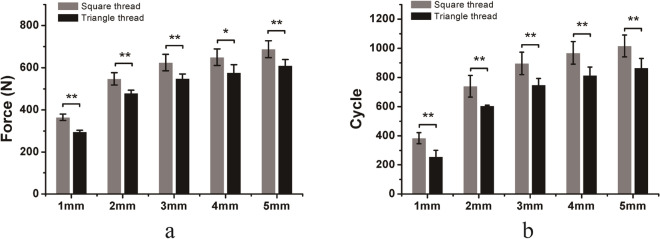
Comparison of the force (**a**) and number of cycles (**b**) required to achieve a 1, 2, 3, 4, and 5 mm displacement of the locking screws (LP fixation) with square thread and triangle thread under cyclic craniocaudal loading. Mean values ± standard deviation of the measured values are presented (N = 5) (Student’s t-test, *p < 0.05, **p < 0.01).

For LP/locking screw constructs under craniocaudal loading, locking screws with square threads required higher load and more cycles to reach the same displacement than locking screws with triangle threads. The differences in load and number of cycles required to achieve a 1, 2, 3, 4, and 5 mm displacement of the screws with two different thread types were significant (Fig. 5). In general, locking screws with square threads had better fixation stability than those with triangle threads under craniocaudal loading.

**Figure 6:**
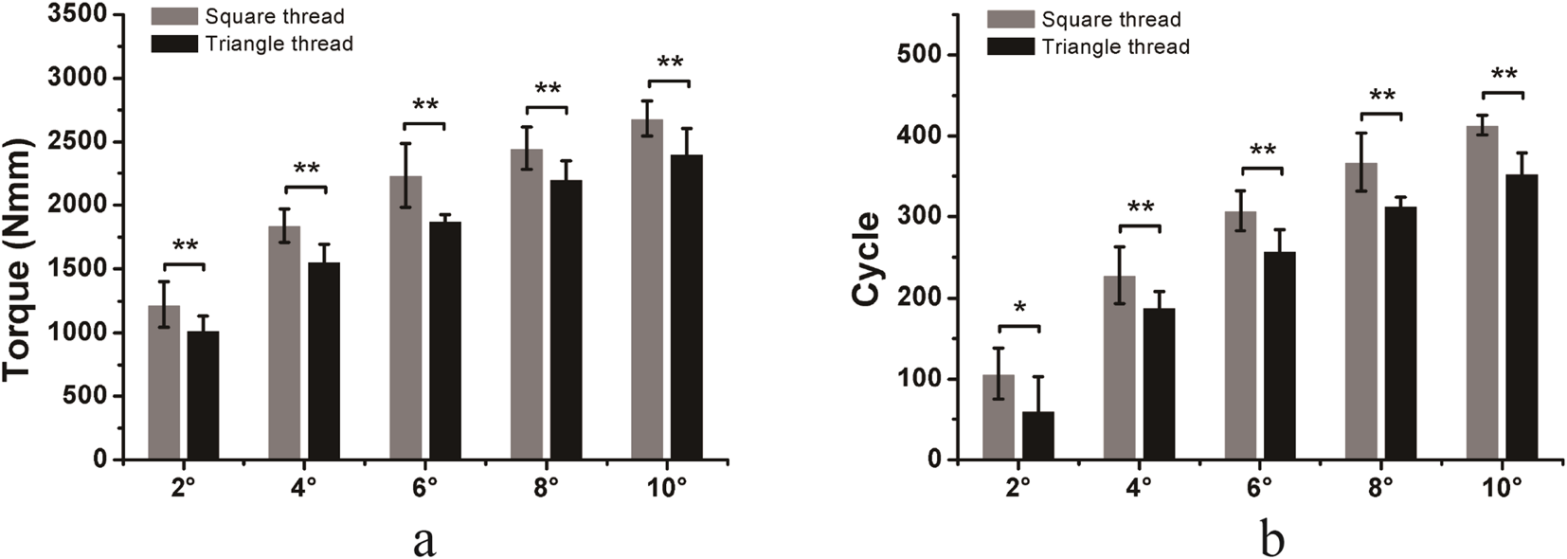
Comparison of torque (**a**) and number of cycles (**b**) required to achieve 2, 4, 6, 8, and 10 degrees of torsion of the locking screws (LP fixation) with square threads and triangle threads under cyclic torsional loading. Mean values ± standard deviation of the measured values are presented (N = 5) (Student’s t-test, *p < 0.05, **p < 0.01).

For LP/locking screw constructs under torsional loading, locking screws with square threads required higher torque and more cycles to reach the same angle of torsion than triangle threaded locking screws. The differences in torque and number of cycles required to achieve 2, 4, 6, 8, and 10 degrees of torsion in the two different thread types were significant (Fig. 6). In general, locking screws with square threads had better fixation stability than those with triangle threads under torsional loading.

**Figure 7:**
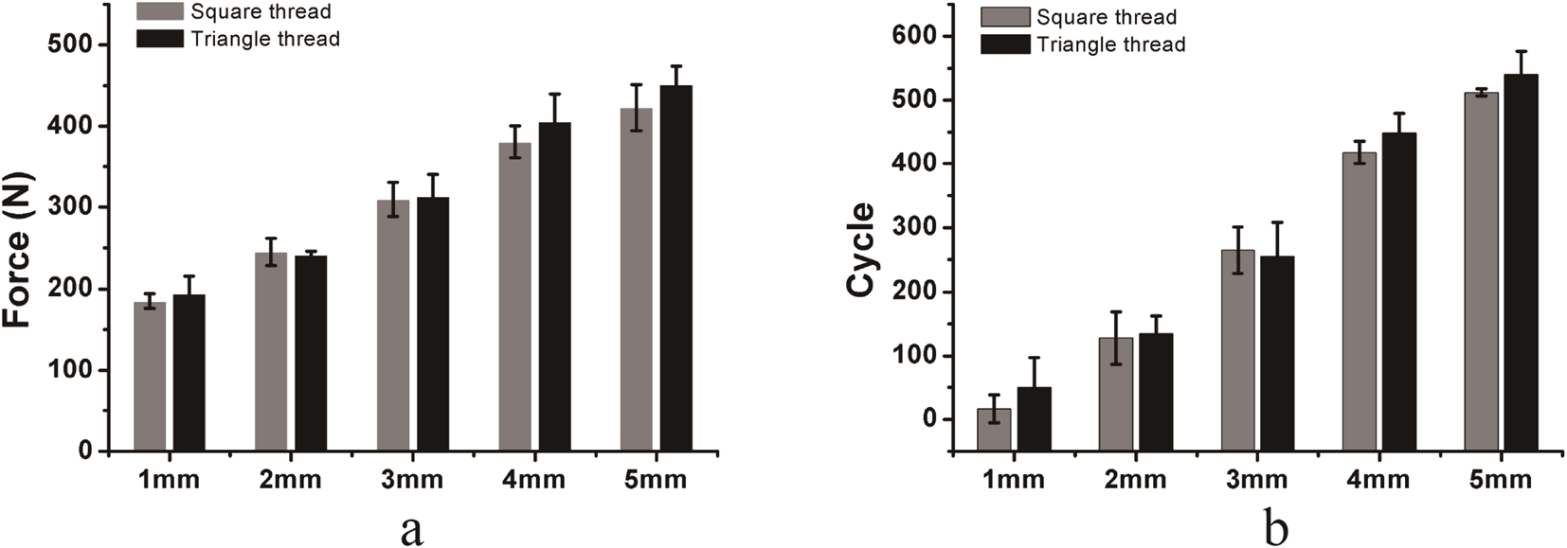
Comparison of the force (**a**) and number of cycles (**b**) required to achieve a 1, 2, 3, 4, and 5 mm displacement of the compression screws (DCP fixation) with square threads and triangle threads under cyclic craniocaudal loading. Mean values ± standard deviation of the measured values are presented (N = 5) (Student’s t-test, *p < 0.05, **p < 0.01).

For DCP/compression screw constructs under craniocaudal loading, the load and number of cycles required to reach the same displacement for the two screw types were similar, and no significant differences were found (Fig. 7). In general, compression screws with square threads had comparable fixation stability to those with triangle threads under craniocaudal loading.

**Figure 8:**
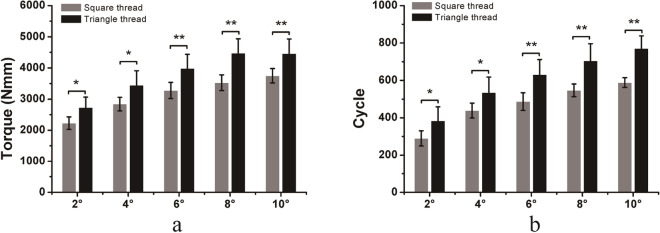
Comparison of the torque (**a**) and number of cycles (**b**) required to achieve 2, 4, 6, 8, and 10 degrees of torsion of the compression screws (DCP fixation) with square threads and triangle threads under cyclic torsional loading. Mean values ± standard deviation of the measured values are presented (N = 5) (Student’s t-test, *p < 0.05, **p < 0.01).

For DCP/compression screw constructs under torsional loading, compression screws with triangle threads required higher torque, and more cycles to reach the same angle of torsion than those with square threads. The differences in torque and number of cycles required to achieve 2, 4, 6, 8, and 10 degrees of torsion in the two different thread types were significant (Fig. 8). In general, compression screws with triangle threads had better fixation stability than those with square threads under torsional loading.

### Association between individual screw characteristics and stability of plate fixation

Locking screws with superior lateral migration resistance had better fixation stability when applied to a locking plate, while axial pullout strength of locking screws showed no association with fixation stability under both cyclic craniocaudal loading and torsional loading.

Compression screws with superior lateral migration resistance or axial pullout strength did not show better fixation stability under cyclic craniocaudal loading. Compression screws with superior axial pullout strength had better fixation stability when applied to a dynamic compression plate, while lateral migration resistance of compression screws showed no association with fixation stability under torsional loading.

## Discussion

Evaluation of the fixation stability of bone screws often focuses on simple axial pullout, which rarely simulates physiological loading conditions, while lateral migration resistance, another important biomechanical characteristic for bone screws, has been neglected^10–15^. Axial pullout strength and lateral migration resistance of individual screws correspond to forces which are perpendicular to each other, which means these two characteristics are independent and mutually non-interacting. Therefore, axial pullout strength and lateral migration resistance were used as the two biomechanical characteristics of individual screws measured in this study. The purpose of testing the biomechanical characteristics of different individual screws was to identify appropriate screws for the plate fixation test (i.e., those with the highest axial pullout resistance and lateral migration resistance respectively). Since different thread types alter the contact surface area with the bone, and thus the pullout strength and lateral migration resistance of the screw^15,31–34^, this study used identical screws that only differed in thread type (buttress, square, or triangle thread). The result of pullout test in this study was consistent with the finding of a biomechanical pullout test performed by Kim that triangle shape thread showed higher pullout strength than buttress thread and square thread pedicle screws in synthetic cancellous bone^34^.

The experimental design in this study enabled us to clarify the association between a screw’s biomechanical characteristics and its stability during plate fixation to determine which screw characteristic is the primary contributor to fixation stability when applied to a plate. In the LP/locking screw constructs, screws with superior lateral migration resistance had better fixation stability under both cyclic craniocaudal loading and torsional loading; however, screws with superior axial pullout strength did not have better fixation stability under either type of loading. This suggests that fixation stability of the locking screw is directly related to its lateral migration resistance, while axial pullout strength is not involved in resisting cyclic craniocaudal or torsional loading. This is consistent with the finding of Kueny et al. in a pedicle screw biomechanical study, in which the difference in axial pullout strength among different pedicle screws was not reflected in the more physiologically relevant fatigue testing^20^; however, they did not consider lateral migration resistance as an influencing factor of screw fixation stability as we did in our study.

In the DCP/compression screw constructs, screws with superior lateral migration resistance but inferior axial pullout strength had similar fixation stability under cyclic craniocaudal loading to screws with superior axial pullout strength but inferior lateral migration resistance. This indicates that, in compression screws, both lateral migration resistance and axial pullout strength are working together to resist cyclic craniocaudal loading. Because compression screws are not locked with the screw hole of the plates, when craniocaudal loading is applied to the bone, the plates oppositely push the screws’ head upward so that the whole screw has a trend of unilateral migration and axial pullout. Screws with superior axial pullout strength, but not those with superior lateral migration resistance, had better fixation stability under torsional loading. This suggests that axial pullout strength is the primary contributor to fixation stability of the compression screw, while lateral migration resistance is not involved in resisting torsional loading.

Axial pullout tests have been commonly used as the standard test to predict the fixation strength of medical bone screws. According to the results of this study, screws with good axial pullout resistance do not necessarily have better fixation stability. Axial pullout tests can only be used to predict the axial pullout strength of a screw, not its fixation stability, which suggests that an axial pullout test alone is insufficient to evaluate the fixation stability of a screw. The lateral migration test described in this study is simple and repeatable and should be an important factor in bone screw evaluation. This study underscores the importance of using different tests to evaluate different screw types under different loading conditions. When evaluating the craniocaudal and torsional loading stability of a locking screw, a lateral migration test should be used. When evaluating the craniocaudal loading of a compression screw, both a lateral migration and axial pullout test should be used. When evaluating the torsional loading stability of a compression screw, an axial pullout test should be used.

This study attempted to elucidate the fixation stability of different screw types under two specific loads on long bones. Cyclic craniocaudal loading and torsional loading were used to simulate the normal physiological loads of humans during daily activity^28^. For cyclic craniocaudal loading, we started with 100–200 N and increased the load by 50 N every 100 cycles to simulate the weight bearing of walking activity experienced by the lower extremities. A similar loading pattern was used in a study of pedicle screw fixation stability^35^. For torsional loading, we started with ± 1 Nm and increased by 0.5 Nm every 100 cycles to simulate the torque loading of a long bone during daily activity. The 1 Nm starting value was chosen because it is 10% of the maximum torque that the 30 × 30 × H60 mm polyurethane foam blocks could withstand, as tested in a pilot study. Clinically, screw loosening is defined as a radiolucency more than 1 mm surrounding the screw^36^. However, most clinical cases show no significant clinical consequences to a 1 mm radiolucency^6^. In this study, 5 mm and 10 degree endpoints were selected to evaluate the fixation stability of the screws at significant failure. The load and number of cycles required to achieve 1, 2, 3, 4, and 5 mm displacement and the torque and number of cycles required to achieve 2, 4, 6, 8 and 10 degree torsion were recorded to quantify the fixation stability of the screws.

There are limitations to this study. First, biomechanical studies with polyurethane foam blocks cannot fully replicate in vivo conditions and therefore, such limitations must be considered when attempting to draw conclusions. While synthetic bone models are a consistent medium for mechanical testing, they do not perfectly simulate normal human bone. What is more, the polyurethane foam models used in this study have previously been accepted as a substitute for cadaveric bone in biomechanical testing^24–26^. The polyurethane foam is microstructurally similar to human bone and exhibits similar mechanical properties while avoiding inconsistencies due to interindividual variations in human bones. Second, this study used cyclic craniocaudal loading and torsional loading to simulate physiological loading, which cannot fully replicate the highly complex loading conditions of human arms and legs during daily activity. However, the loadings used in this study are already able to simulate most daily activity loading. Third, simulated bone with normal density was tested in this biomechanical study as a representative. Bone densities representing osteopenia and osteoporosis will be tested in a further study to strengthen the conclusion of this study. Fourth, the use of two-screws-per-segment fixation instead of using three screws and the use of polyurethane specimens without a cortical shell were unable to represent the real clinical environment. However, the choice of such experimental model allowed us to recreate under in vitro conditions the worst possible clinical situation, in which the bone could not hold the screws firmly, to better evaluate the fixation stability of different screw designs. Finally, because of the unique design of this study, the accompanying underpowered nature of the study should be considered. The conclusions of this study are drawn considering these limitations.

## Conclusion

This biomechanical study demonstrates that lateral migration resistance of a locking screw is the primary contributor to fixation stability in resisting both craniocaudal and torsional loading. For compression screws, lateral migration resistance and axial pullout strength work together to resist cyclic craniocaudal loading, while the ability to resist torsional loading is mainly provided by axial pullout strength.
